# Sulfate Exchange of the Nitrate-Type Layered Hydroxide Nanosheets of Ln_2_(OH)_5_NO_3_·*n*H_2_O for Better Dispersed and Multi-color Luminescent Ln_2_O_3_ Nanophosphors (Ln = Y_0.98_RE_0.02_, RE = Pr, Sm, Eu, Tb, Dy, Ho, Er, and Tm)

**DOI:** 10.1186/s11671-016-1544-0

**Published:** 2016-07-12

**Authors:** Xiaoli Wu, Weigang Liu, Ji-Guang Li, Qi Zhu, Xiaodong Li, Xudong Sun

**Affiliations:** Key Laboratory for Anisotropy and Texture of Materials (Ministry of Education), School of Materials Science and Engineering, Northeastern University, Shenyang, Liaoning 110819 China; Materials Processing Unit, National Institute for Materials Science, Tsukuba, Ibaraki 305-0044 Japan; College of Material Science and Engineering, Guilin University of Technology, Guilin, Guangxi 541004 China

**Keywords:** Nanosheets, Anion exchange, Phase evolution, Oxide phosphor, Luminescence

## Abstract

Through restricting thickness growth by performing coprecipitation at the freezing temperature of ~4 °C, solid-solution nanosheets (up to 5-nm thick) of the Ln_2_(OH)_5_NO_3_·*n*H_2_O layered hydroxide (Ln = Y_0.98_RE_0.02_; RE = Pr, Sm, Eu, Tb, Dy, Ho, Er, and Tm, respectively) were directly synthesized without performing conventional exfoliation. In situ exchange of the interlayer NO_3_^−^ with SO_4_^2−^ produced a sulfate derivative [Ln_2_(OH)_5_(SO_4_)_0.5_·*n*H_2_O] of the same layered structure and two-dimensional crystallite morphology but substantially contracted *d*_002_ basal spacing (from ~0.886 to 0.841 nm). The sulfate derivative was systematically compared against its nitrate parent in terms of crystal structure and phase/morphology evolution upon heating. It is shown that the interlayer SO_4_^2−^, owing to its bonding with the hydroxide main layer, significantly raises the decomposition temperature from ~600 to 1000 °C to yield remarkably better dispersed oxide nanopowders via a monoclinic Ln_2_O_2_SO_4_ intermediate. The resultant (Y_0.98_RE_0.02_)_2_O_3_ nanophosphors were studied for their photoluminescence to show that the emission color, depending on RE^3+^, spans a wide range in the Commission Internationale de l’Eclairage (CIE) chromaticity diagram, from blue to deep red via green, yellow, orange, and orange red.

## Background

Y_2_O_3_ is a widely used host lattice in the phosphor field, owing to its excellent structure stability, chemical durability, and particularly its ability to accept a substantial amount of various trivalent rare-earth activators for a broad range of optical functionalities. Due to their technical importance in the lighting and display areas, Y_2_O_3_-based phosphors are being widely investigated to correlate their luminescent performance with the characteristics of the phosphor powder [1, 2]. Controllable synthesis has always been an active area of phosphor study, and the well-adopted processing technologies may include flux-assisted solid state reaction [3, 4], solution synthesis, combustion [5], spray pyrolysis [6–8], and gas-phase condensation [9, 10].

Among the aforementioned synthetic strategies, solution processing is of particular interest since it allows a facile manipulation of particle morphology. With this technique, Y_2_O_3_-based phosphors that have the various morphologies of zero-dimensional (0D) nanoparticles, monodispersed microspheres [11, 12], 1D nanowires/nanotubes [13–15], 2D nanoplates [16–18], and hierarchical structures [2, 19] have been obtained. As the direct product of solution synthesis is usually a precursor, the final phosphor oxide is thus frequently observed to have properties dependent on the characteristics of its precursor [2]. Layered rare-earth hydroxide (LRH), as a relatively new type of anionic layered compounds [20], has attracted much attention during the recent years owing to its unique combination of the layered structure and the abundant optical, magnetic, and catalytic properties of the rare-earth elements [21–34]. The crystal structure of Ln_2_(OH)_5_A·*n*H_2_O LRH (Ln = rare-earth; *A* = NO_3_^−^ or halogen anion; *n* ~ 1.5) can be viewed as an alternative stacking along the *c*-axis ([001] direction) of the positively charged hydroxide main layers containing Ln^3+^ and exchangeable A anions located in the interlayer for charge balance. The well-established synthetic methodologies of hydrothermal reaction [25–34] and reflux growth [21–24] generally produce platelike LRH crystals of several microns in lateral dimension and tens to hundreds of nanometers in thickness, for which single layer or few-layer thick nanosheets can only be obtained by swelling the pristine crystals via exchange of the interlayer anions with significantly larger ones (such as dodecyl sulfate, DS^−^), followed by exfoliation in a proper medium (such as formamide) under mechanical agitation [35–39]. Exfoliation, however, is well known to be time consuming, frequently incomplete, and usually accompanied by fragmentation of nanosheets. We previously reported a capped growth technique to synthesize nanometer-thin LRH flakes via one-step hydrothermal reaction [32], but the batch yield is rather limited. Both the pristine LRH crystals and the exfoliated nanosheets can serve as new precursors for oxide phosphors and phosphor films [35–39], but thick crystallites would not collapse into nanoparticles via calcination and the resultant oxides frequently retain platelike morphologies [40–42].

The hydroxide main layer of LRH is a close-packed low-energy plane, and thus, its two-dimensional growth needs lower activation energy than the thickness growth along the [001] direction. We recently demonstrated that, through suppressing thickness growth by lowering the synthesis temperature to ~4 °C, NO_3_^−^-LRH nanosheets of only ~4-nm thick can be directly crystallized, without exfoliation, for a wide spectrum of single Ln (Ln = Pr-Er, and Y) [43]. With this technique, similarly thin nanosheets were produced in this work for the LRH solid solutions of Y/RE (RE = Pr, Sm, Eu, Tb, Dy, Ho, Er, and Tm) in good batch quantity (0.03 mol of LRH or ~10 g). The effects of SO_4_^2−^ exchange for interlayer NO_3_^−^ on crystal structure and thermal behavior of the nanosheets and also characteristics and luminescent properties of the derived (Y_0.98_RE_0.02_)_2_O_3_ nanophosphors were studied in detail.

## Methods

### Freezing Temperature Crystallization of LRH Solid-Solution Nanosheets

The starting rare-earth sources are Pr_6_O_11_ (99.96 % pure), Tb_4_O_7_ (99.99 % pure), and RE_2_O_3_ (99.99 % pure, RE = Y, Sm, Eu, Dy, Ho, Er, and Tm), all were purchased from Huizhou Ruier Rare-Chem. Hi-Tech. Co. Ltd (Huizhou, China). The other reagents of ammonium hydroxide solution (25 %), nitric acid (63 wt.%), and ammonium sulfate are of analytical grade and were purchased from Shenyang Chemical Reagent Factory (Shenyang, China). Nitrate solution of the rare earth was prepared by dissolving the oxide with a proper amount of nitric acid, followed by evaporation to dryness at 95 °C to remove superfluous HNO_3_ and a final dilution to 1.0 mol/L.

In a typical synthesis, a diluted ammonium hydroxide solution (1.0 mol/L) was slowly dripped (~2.0 mL/min) into 300 mL of a 0.2 mol/L nitrate solution of Ln^3+^ (Ln = Y_0.98_RE_0.02_) kept at ~4 °C until pH ~8 to produce Ln_2_(OH)_5_NO_3_·*n*H_2_O nanosheets (30 mmol of Ln_2_(OH)_5_NO_3_·*n*H_2_O per batch) [43]. For anion exchange with SO_4_^2^−100 mL aqueous solution containing 15 mmol of (NH_4_)_2_SO_4_ (one SO_4_^2−^ would replace two NO_3_^−^) was added into the nanosheets suspension after 20 min of magnetic stirring (in situ anion exchange). The final product was collected via centrifugation after reaction for 1 h, followed by sequential washing with distilled water three times and ethanol one time and then air drying at 50 °C for 15 h. Calcination of the dried nanosheets was performed in flowing oxygen gas (200 mL/min), using a heating rate of 5 °C/min at the ramp stage and a holding time of 4 h. The final phosphor powders are all calcined at 1100 °C, but with the Pr- and Tb-containing samples being subjected to an additional reduction in flowing H_2_ (200 mL/min) at 1100 °C for 1 h.

### Characterization Techniques

Chemical analysis of the products was performed for Ln via inductively coupled plasma (ICP) spectroscopy (Model IRIS Advantage, Jarrell-Ash Japan, Kyoto), for NO_3_^−^ via spectrophotometry (Ubest-35, Japan Spectroscopic Co., Ltd., Tokyo), and for S via combustion-infrared absorptiometry (Model CS-444LS, LECO, St. Joseph, MI). The detection limits of these analyses are all 0.01 wt.%. Phase identification was made via X-ray diffractometry (XRD; Model PW3040/60, Panalytical B.V., Almelo, the Netherlands) operated at 40 kV/40 mA, using nickel-filtered Cu-*K*α radiation (*λ* = 0.15406 nm) and a scanning speed of 1.0° 2*θ*/min. Fourier transform infrared spectroscopy (FTIR; Model Spectrum RXI, Perkin-Elmer, Shelton, CT) was performed by the standard KBr method. Powder morphology was analyzed by transmission electron microscopy under an acceleration voltage of 200 kV (TEM; Model JEM-2000FX, JEOL, Tokyo) and field emission scanning electron microscopy (FE-SEM; Model S-5000, Hitachi, Tokyo) under 10 kV. Thermogravimetry (TG; Model 8120, Rigaku, Tokyo) of the nanosheets was conducted in flowing air (100 mL/min) with a constant heating rate of 10 °C/min. Specific surface area of the oxide phosphor was obtained with an automatic analyzer (Model TriStar II 3020, Micromeritics Instrument Corp., Norcross, GA) using the Brunauer-Emmett-Teller (BET) method via nitrogen adsorption at 77 K. Particle size/size distribution analysis was made with a laser-diffraction particle sizer (Model LA-920, Horiba Scientific, Kyoto), after ultrasonically dispersing the oxide powder in ethanol. Photoluminescence was analyzed at room temperature using an FP-6500 fluorospectrophotometer (JASCO, Tokyo) equipped with a 60-mm-diameter integrating sphere (Model ISF-513, JASCO) and a 150-W Xe-lamp for excitation. Measurements were conducted under identical conditions for all the samples, using a scan speed of 100 nm/min and slit widths of 5 nm for both excitation and emission. Fluorescence lifetime of the luminescence was analyzed with the FP-6500 equipment for Sm^3+^, Eu^3+^, and Tb^3+^, and with a DeltaFlex lifetime fluorescence spectrometer (Horiba Scientific) for the fast decay of Pr^3+^, Dy^3+^, Ho^3+^, Er^3+^, and Tm^3+^.

## Results and Discussion

### Characteristics of the NO_3_^−^-LLnH Nanosheets and the Effects of SO_4_^2−^ Exchange

**Fig. 1:**
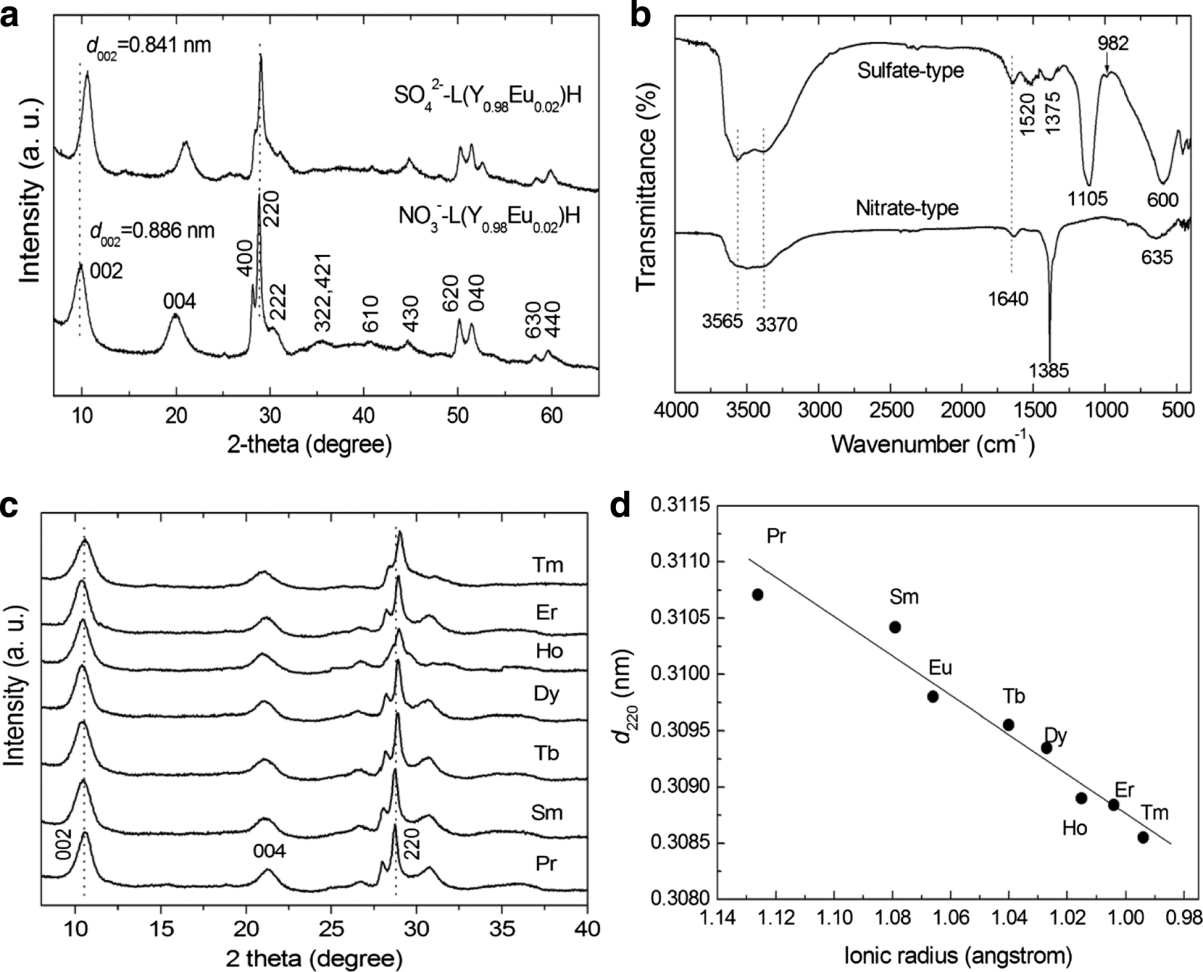
Powder XRD patterns and FT-IR spectra of the products and the 220 spacing (*d*_220_) of the SO_4_^2−^-L(Y_0.98_RE_0.02_)H. **a** Powder XRD patterns and **b** FT-IR spectra of the NO_3_^−^-L(Y_0.98_Eu_0.02_)H and SO_4_^2−^-L(Y_0.98_Eu_0.02_)H samples. Part **c** displays XRD patterns of the rest SO_4_^2−^-L(Y_0.98_RE_0.02_)Hs synthesized in this work, and part **d** is the 220 spacing (*d*_220_) of the SO_4_^2−^-L(Y_0.98_RE_0.02_)H, as a function of the ionic radius of the RE^3+^ dopant (taken for CN = 8)

The effects of SO_4_^2−^ exchange on the crystal structure of LLnHs were analyzed with L(Y_0.98_Eu_0.02_)H for example. Figure 1a compares XRD patterns of the NO_3_^−^-L(Y_0.98_Eu_0.02_)H and its SO_4_^2−^ derivative. The NO_3_^−^-type exhibits the characteristic 00*l* and non-00*l* diffractions of Ln_2_(OH)_5_NO_3_·*n*H_2_O layered compounds [20–34]. The strong and sharp 220 diffraction, arising from the *ab* plane (the hydroxide main layer), suggests that the host layers of L(Y_0.98_Eu_0.02_)H are well crystallized. Compared with the thick LRH crystals synthesized under high temperature [21–31, 33, 34], the 002 diffraction has a substantially lower intensity relative to the 220 one, implying that the primary crystallites are much less developed along the *c*-axis or rather thin, as also confirmed later by TEM analysis. SO_4_^2−^ exchange of NO_3_^−^ shortens the interlayer distance, as perceived from the obvious shifting of the 00*l* diffraction to a higher angle, and the basal spacing (*d*_002_) calculated from the center of the 002 peak is ~0.886 nm for NO_3_^−^-L(Y_0.98_Eu_0.02_)H and 0.841 nm for the SO_4_^2−^ derivative. The values are close to those found for NO_3_^−^-LYH and its exchange product, respectively [43]. The sulfate derivative still exhibits quite strong 220 diffraction, indicating that sulfate exchange did not appreciably damage the hydroxide main layers. Shifting of the 220 peak from 2*θ* ~ 28.86° to 29.02° and decreased intensity of the 400 diffraction by the anion exchange, however, suggests that the intercalated SO_4_^2−^ is interacting with the hydroxide layers to deteriorate crystallinity of the sample. The interaction, mostly through hydrogen bonding with the hydroxyls/H_2_O in the [Ln(OH)_7_H_2_O] and [Ln(OH)_8_H_2_O] polyhedrons that comprise the hydroxide layers [21–24], leads to lattice distortion and thus the slight peak-shifting [43]. The hydrogen bonding is also responsible for the observed interlayer contraction, since it would draw closer the adjacent positively charged hydroxide layers [43].

**Fig. 2:**
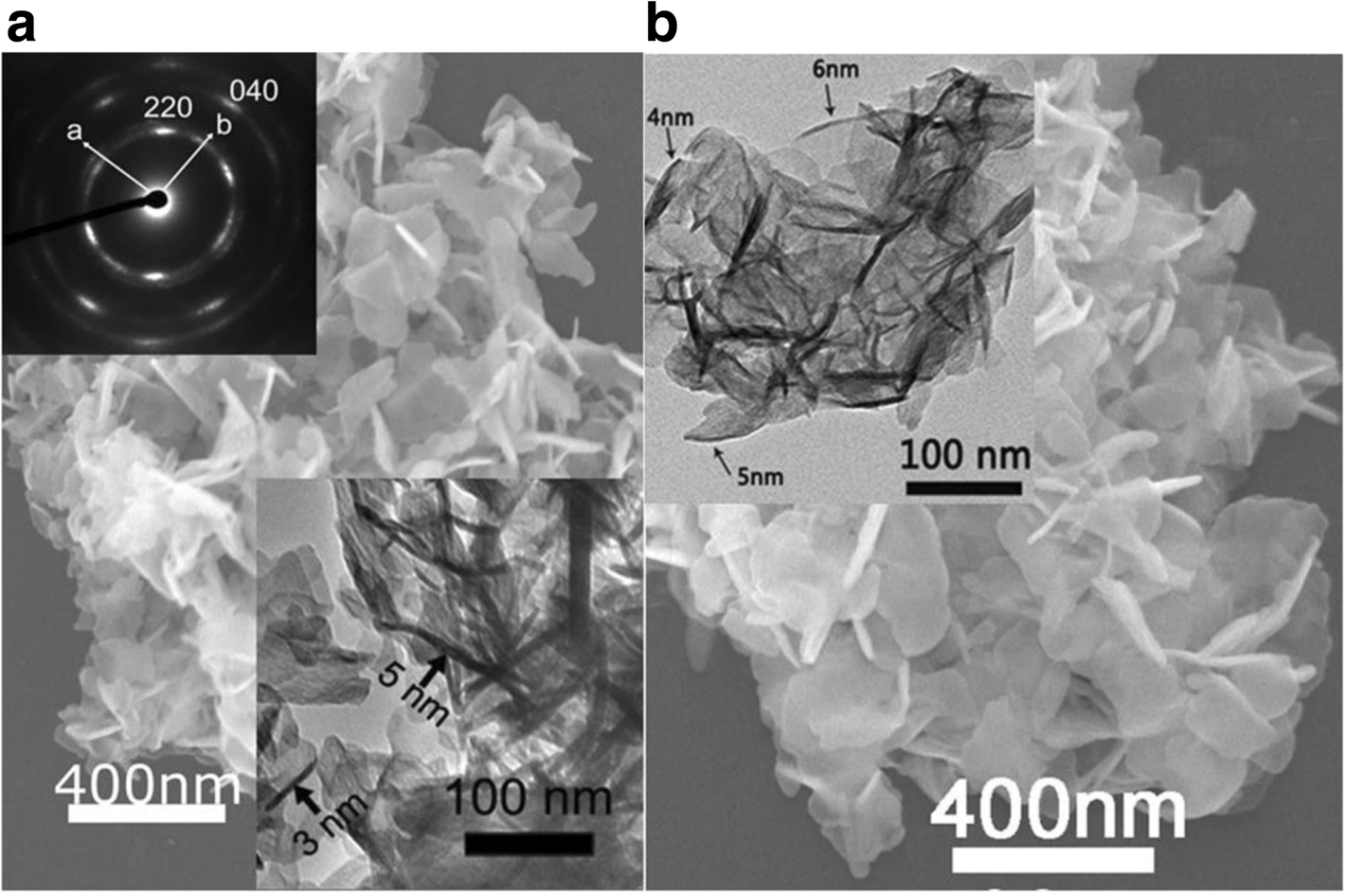
SEM and TEM morphology of the products. SEM micrographs showing overall morphologies of the NO_3_^−^-L(Y_0.98_Eu_0.02_)H (**a**) and SO_4_^2−^-L(Y_0.98_Eu_0.02_)H (**b**) samples. The *insets* are the TEM morphology and selected area electron diffraction (SAED) pattern of the nanosheets

Chemical analysis of the NO_3_^−^-L(Y_0.98_Eu_0.02_)H found ~1.65 wt.% of Eu, 49.88 wt.% of Y, and 16.68 wt.% of NO_3_^−^, corresponding to Y/Eu and NO_3_^−^/Ln (Ln = Y and Eu) molar ratios of 0.98/0.019 and 0.94/2 (close to 1/2), respectively. The results thus confirm that the prescribed Y/Eu atomic ratio (0.98/0.02) has essentially been kept to the product and NO_3_^−^-L(Y_0.98_Eu_0.02_)H has been formed. The SO_4_^2−^ exchange product was found to have ~1.87 wt.% of Eu, 51.17 wt.% of Y, 4.50 wt.% of S, and trace NO_3_^−^(0.18 wt.%), which lead to Y/Eu and SO_4_^2−^/Ln molar ratios of 0.98/0.021 and 0.48/2 (close to 0.5/2), respectively. The outcomes thus indicate that the anion exchange did not appreciably alter the Y/Eu ratio, and SO_4_^2−^ exchange of the interlayer NO_3_^−^ is virtually complete. The results of the chemical analysis conform to those of FTIR spectroscopy (Fig. 1b). It is clearly seen that the intense NO_3_^−^ absorption at ~1385 cm^−1^ (ν_3_ vibration, as of free anion) vanished, and meanwhile the ν_3_ (~1105 cm^−1^) and ν_1_ (~982 cm^−1^) absorptions, being characteristic of SO_4_^2−^, appeared from the exchange product. The non-splitting feature of ν_3_ suggests that SO_4_^2−^ is not directly coordinated to the metal center in the hydroxide layer while the emergence of ν_1_ implies that the SO_4_^2−^ tetrahedron is distorted owing to the effects of hydrogen bonding [43–45]. It is also owing to the effects of hydrogen bonding that the stretching vibrations of hydroxyls (~3565 cm^−1^) and the O–H radicals in hydration water (~3370 cm^−1^) are both substantially enhanced [44, 45]. The twin absorption bands at ~1520 and 1375 cm^−1^ indicate contamination of the product by CO_3_^2−^, mostly from dissolved atmospheric CO_2_ during synthesis. All the SO_4_^2−^-LLnHs made in this work show almost identical interlayer distances owing to the limited content of RE, but the 220 diffraction successively shifts towards a higher angle along with the decreasing ionic size of the RE^3+^ dopant (Fig. 2c). The 220 spacing (*d*_220_) is shown in Fig. 2d as a function of RE^3+^ size (for eightfold coordination) [46]. As the 220 diffraction reflects metal-to-metal distance in the hydroxide layer [21–24], the *d*_220_ value thus monotonically decreases towards a smaller RE^3+^ as expected. The results also provide direct evidence of solid-solution formation.

Figure 2 shows the results of electron microscopy for the two types of L(Y_0.98_Eu_0.02_)Hs. FE-SEM observation found that the NO_3_^−^-L(Y_0.98_Eu_0.02_)H is composed of 3D flower-like assemblies of nanoflakes having lateral dimensions up to ~300 nm (Fig. 2a, coated with 10-nm-thick tungsten for electrical conduction), while TEM analysis found entangled nanosheets of up to ~5-nm thick (the inset). Calculated from the *d*_002_ basal spacing of ~0.886 nm, each single nanosheet would have only ~5–6 stacking repetitions along the *c*-axis. Selected area electron diffraction (SAED) yielded a well-arranged spot-like pattern (the inset) for the hydroxide layer, indicating that the individual nanosheets are primary of single crystalline and are well crystallized. Anion exchange with SO_4_^2−^ did not incur any appreciable morphology change to either the overall flower-like assemblies or the individual nanosheets (Fig. 2b), in compliance with our previous observations on NO_3_^−^-LYH [43].

### Decomposition and Phase/Morphology Evolution of the Nanosheets upon Heating

**Fig. 3:**
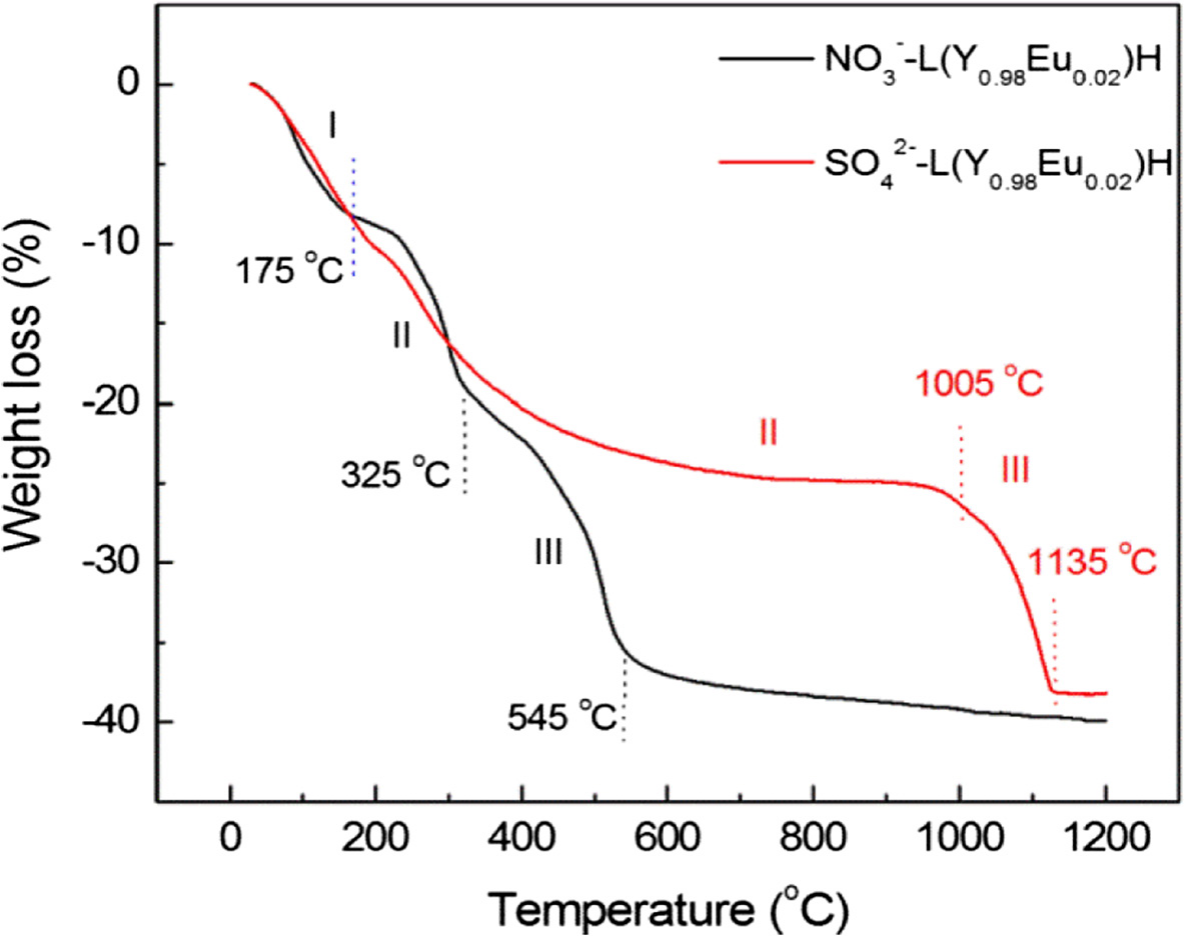
TG curves of NO_3_^−^-and SO_4_^2−^-type L(Y_0.98_Eu_0.02_)Hs. TG traces for the NO_3_^−^- and SO_4_^2−^ type L(Y_0.98_Eu_0.02_)Hs

Thermal behaviors of the nanosheets were analyzed via TG, and the results are compared in Fig. 3 for the NO_3_^−^-L(Y_0.98_Eu_0.02_)H and SO_4_^2−^-L(Y_0.98_Eu_0.02_)H. The NO_3_^−^ type clearly decomposes via three well-defined stages as previously observed for thick LRH crystals [25–29, 32–34], with the first one being dehydration to form Ln_2_(OH)_5_NO_3_ (up to ~175 °C), the second one being dehydroxylation to yield an intermediate mass with nominal composition of (Y_0.98_Eu_0.02_)_2_O_2_(OH)NO_3_ (up to ~325 °C), and the last one being further dehydroxylation and denitration to form oxide (up to ~545 °C). Though the NO_3_^−^-LLnH obtained in this work are nanometer thin, it shows a thermal behavior almost identical to that reported for thick crystals [25–29, 32–34]. The sulfate derivative similarly decomposes via three steps, but only up to the significantly higher temperature of ~1135 °C and with a rather sluggish step in the ~175–1005 °C range (stage II).

**Fig. 4:**
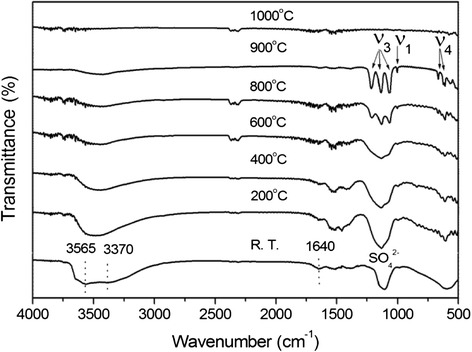
FTIR spectra for the original SO_4_^2−^-L(Y_0.98_Eu_0.02_)H and the calcined products. FTIR spectra for the original SO_4_^2−^-L(Y_0.98_Eu_0.02_)H and the products calcined from it at various selected temperatures for 4 h

To better understand the thermal decomposition of SO_4_^2−^-L(Y_0.98_Eu_0.02_)H, which has not been addressed prior to us, FTIR analysis was performed on the products obtained at various selected temperatures for 4 h (Fig. 4). It is clearly seen that the powders calcined up to 900 °C are characterized by strong SO_4_^2−^ absorptions and successively weaker ones of hydroxyls/water. The results may thus imply that the sluggish weight loss observed on the TG curve from ~175 to 1005 °C is mainly owing to successive dehydroxylation rather than desulfuration. The weight loss (~14.4 %) calculated for dehydroxylation of (Y_0.98_Eu_0.02_)_2_(OH)_5_(SO_4_)_0.5_ to the nominal composition of (Y_0.98_Eu_0.02_)_2_O_2.5_(SO_4_)_0.5_ is indeed close to that found via TG (~16.1 %). It is thus plausible to conclude that the slow dehydroxylation of SO_4_^2−^-L(Y_0.98_Eu_0.02_)H is mainly due to the intramolecular hydrogen bonding between SO_4_^2−^ and OH^−^ groups. Raising the calcination temperature from 900 to 1000 °C simultaneously eliminates the strong SO_4_^2−^ and the already rather weak hydroxyl absorptions, suggesting that the sudden weight loss observed on the TG curve from ~1005 to 1135 °C is dominantly resulted from desulfuration. Again, the weight loss calculated for this thermal event (~14.9 %) is close to the value revealed by TG (~13.5 %). The SO_4_^2−^ anions exhibit significantly split ν_3_ and ν_4_ vibrations for the 800 and 900 °C products, implying that the tetrahedrons of SO_4_^2−^ are substantially distorted by direct coordination to Ln^3+^ [44, 45, 47–49].

**Fig. 5:**
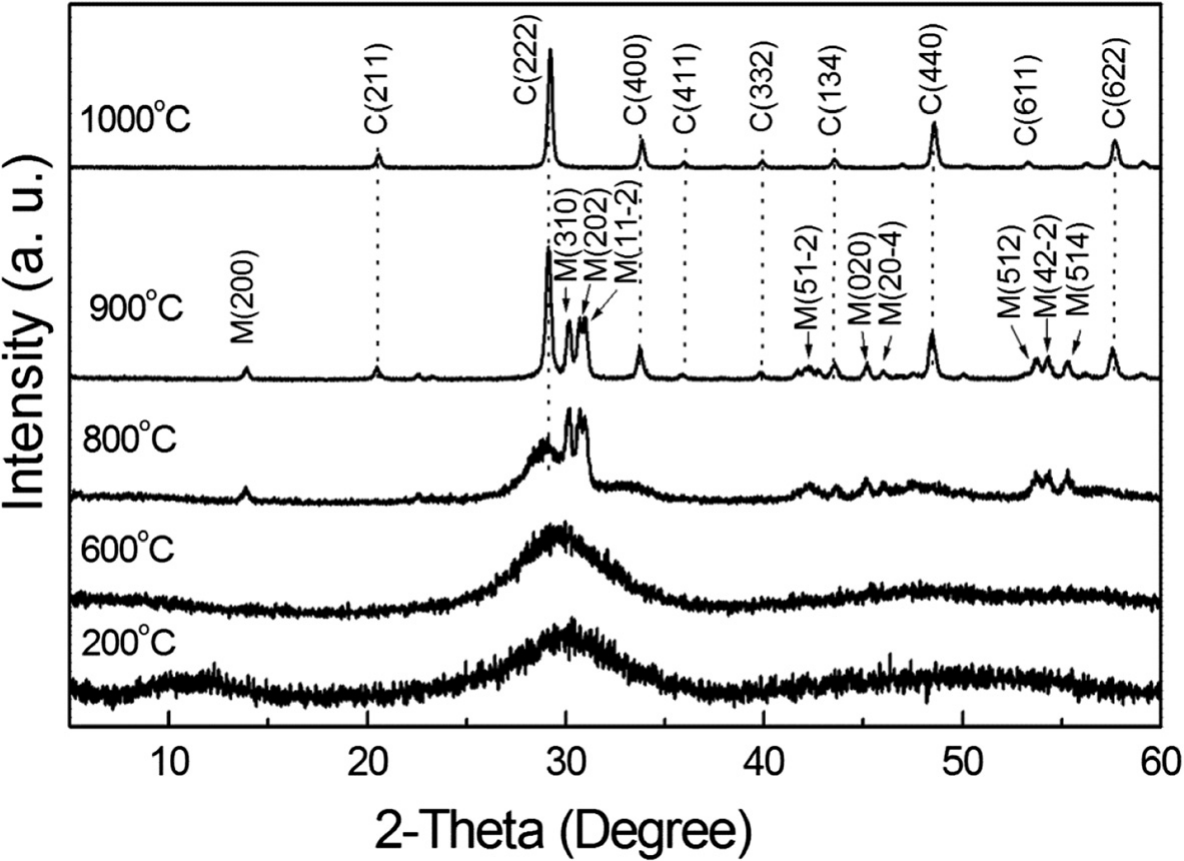
XRD patterns of the products calcined from SO_4_^2−^-L(Y_0.98_Eu_0.02_)H at the different temperatures. XRD patterns of the products calcined from SO_4_^2−^-L(Y_0.98_Eu_0.02_)H at the different temperatures indicated in the figure, where letters *C* and *M* denote cubic (Y_0.98_Eu_0.02_)_2_O_3_ and monoclinic (Y_0.98_Eu_0.02_)_2_O_2_SO_4_ phases, respectively

Phase evolution of the SO_4_^2−^-L(Y_0.98_Eu_0.02_)H upon heating was studied via XRD analysis of the products calcined at different temperatures, and the results are displayed in Fig. 5. It is seen that dehydration of the layered compound at 200 °C leads to an amorphous mass, which persists up to ~600 °C despite the already occurrence of dehydroxylation (Figs. 3 and 4). Further removal of hydroxyls at 800 °C produced a phase mixture of poorly crystallized cubic Ln_2_O_3_ (Ln = Y_0.98_Eu_0.02_, JCPDS: 00-043-1036) and monoclinic Ln_2_O_2_SO_4_ (JCPDS: 00-053-0168), whose diffraction intensities both remarkably improve for the 900 °C product. As the original layered compound has the approximate composition of Ln_2_(OH)_5_(SO_4_)_0.5_·*n*H_2_O, it can thus be said that the 900 °C product is approximately composed of 1/2 mol of Ln_2_O_2_SO_4_ and 1/2 mol of Ln_2_O_3_. Since the SO_4_^2−^ in Ln_2_O_2_SO_4_ is bidentately coordinated to Ln^3+^ [47–49], the significant splitting of the ν_3_ and ν_4_ IR bands was thus observed once the oxy-sulfate compound was formed (Fig. 4). The Ln_2_O_2_SO_4_ component desulfurates at the higher temperature of 1000 °C, and thus, only cubic structured Ln_2_O_3_ was found. The results of XRD comply well with those of FTIR (Fig. 4). That is, the ν_3_ and ν_4_ vibrations of SO_4_^2−^ present as single bands for the 200–600 °C products, as split bands for the 800 and 900 °C products, and vanish for the 1000 °C product. Similar phase evolution analysis of the NO_3_^−^-L(Y_0.98_Eu_0.02_)H found that cubic (Y_0.98_Eu_0.02_)_2_O_3_ crystallizes at ~600 °C via an amorphous state at lower temperatures. The results are not shown here since they are essentially identical to those previously reported by us for thick NO_3_^−^-LRH crystals [33].

**Fig. 6:**
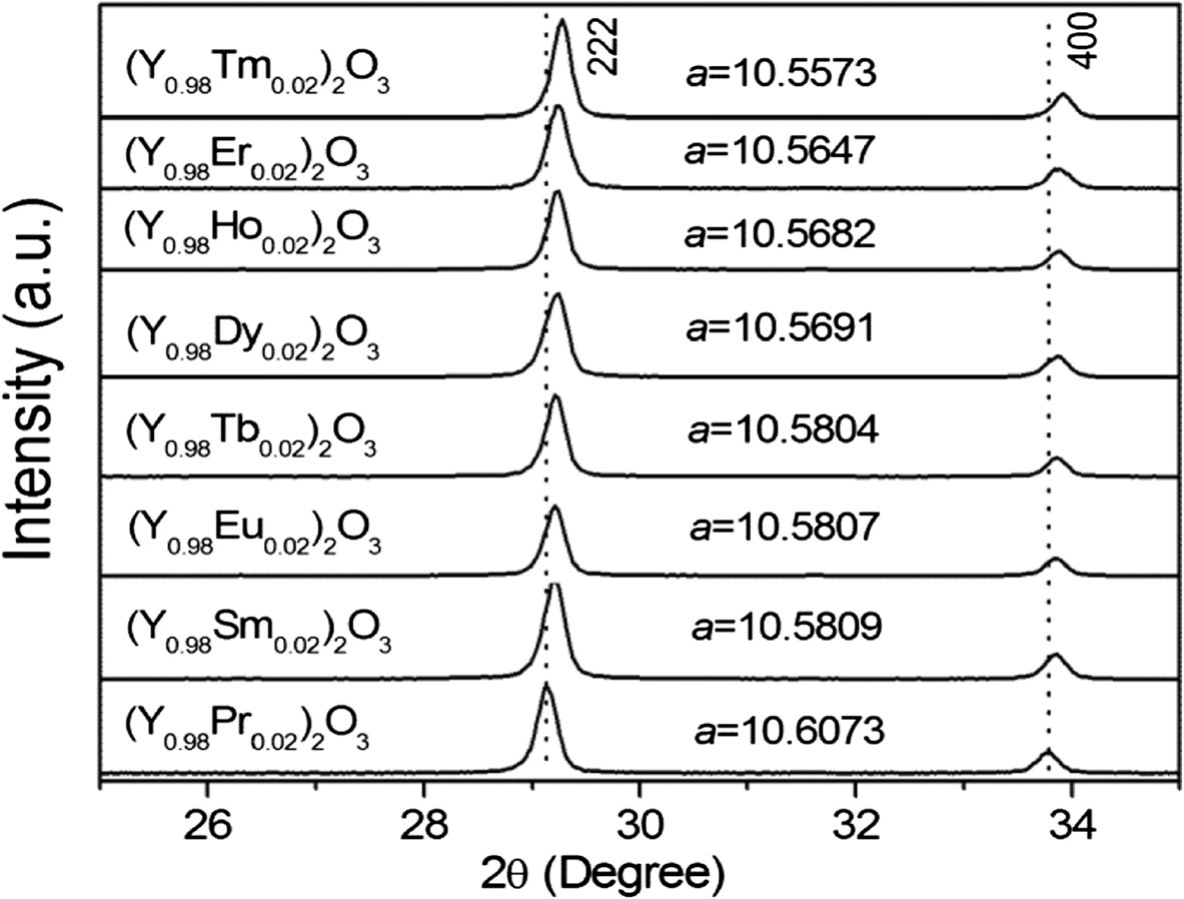
XRD patterns of the (Y_0.98_RE_0.02_)_2_O_3_ powders. XRD patterns of the (Y_0.98_RE_0.02_)_2_O_3_ powders calcined from SO_4_^2−^-L(Y_0.98_RE_0.02_)H at 1100 °C for 4 h, with the cell parameter *a* (in angstrom) included

Figure 6 compares XRD patterns of the oxides calcined from SO_4_^2−^-L(Y_0.98_RE_0.02_)H at 1100 °C for 4 h. Only enlarged view of the 2*θ* = 25–35° region was given to show the effects of RE^3+^ dopant. It is clearly seen that both the (222) and (400) diffractions steadily shift to higher angles along with decreasing ionic radius of RE^3+^, indicating the formation of solid solution. The lattice parameter (*a*, in angstrom) calculated from the strongest (222) diffraction indeed becomes successively smaller towards a smaller RE^3+^ (Fig. 6). The crystallite size assayed from the (222) diffraction by applying Scherrer equation is ~35 nm for all the oxides, irrespective of the dopant type. The cell parameters determined herein are all larger than the 10.547 Å reported for pure Y_2_O_3_ (JCPDS: 00-043-1036, Y^3+^ close to Ho^3+^ in radius), possibly owing to the limited crystallite size of the present powders.

**Fig. 7:**
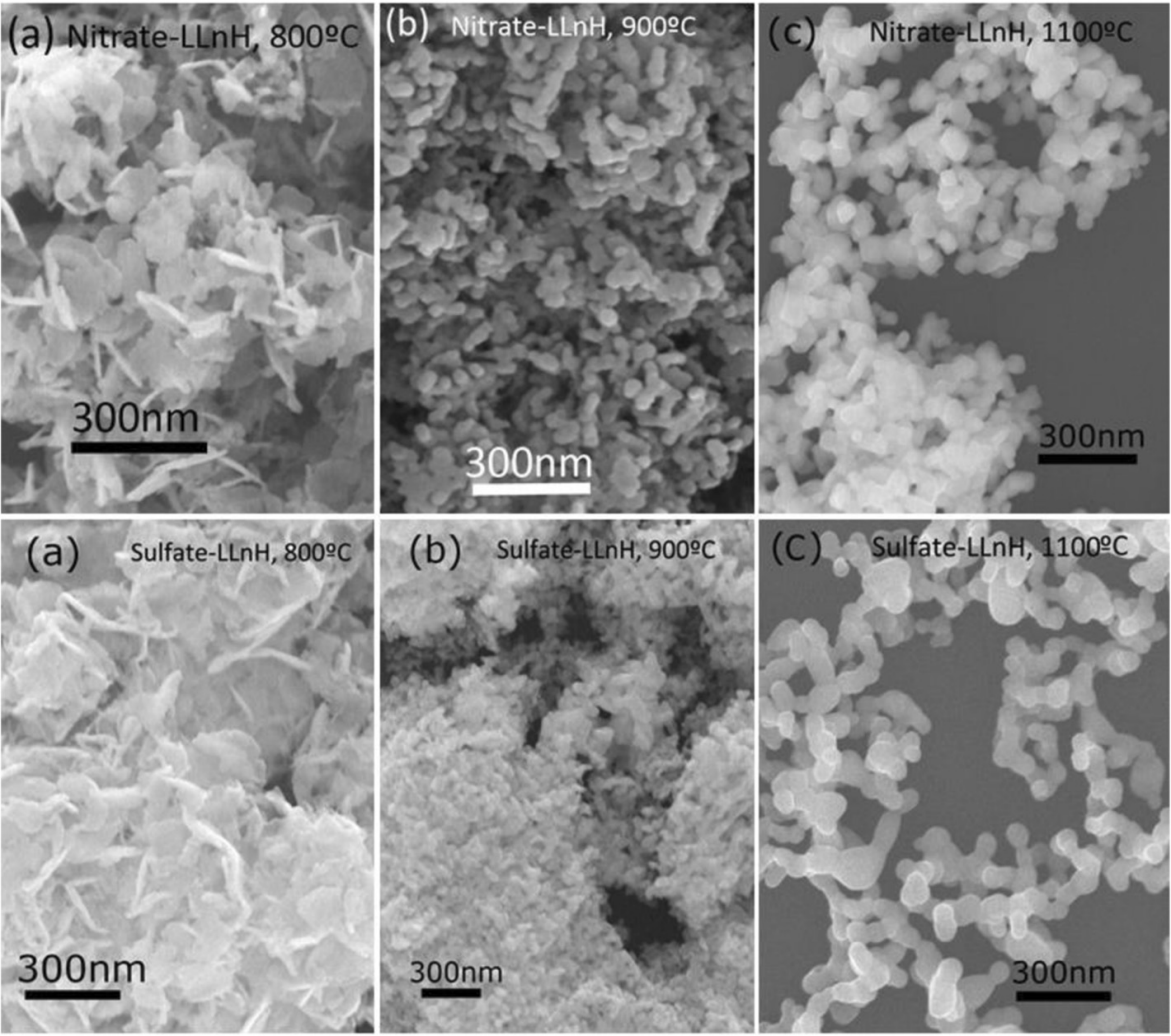
FE-SEM micrographs of the products calcined from NO_3_^−^- and SO_4_^2−^-L(Y_0.98_Eu_0.02_)Hs at the different temperatures. FE-SEM micrographs showing morphologies of the products calcined from NO_3_^−^- and SO_4_^2−^-L(Y_0.98_Eu_0.02_)Hs at the temperatures for (**a**) 800 °C, (**b**) 900 °C and (**c**) 1100 °C

Morphology evolution of the nanosheets during calcination was studied with NO_3_^−^-L(Y_0.98_Eu_0.02_)H and SO_4_^2−^-L(Y_0.98_Eu_0.02_)H for example. Figure 7 exhibits typical FE-SEM morphologies for the powders calcined at some representative temperatures. It is seen that the 800 °C product from NO_3_^−^-L(Y_0.98_Eu_0.02_)H well retained the overall morphology of its precursor, despite that it has been a phase-pure oxide, and the flower-like assemblies (domains) and the individual nanosheets within the domains are clearly observable. Calcination at 900 °C led to substantial collapse of the nanosheets into nanoparticles within each domain, owing to the thermal stress arising from crystallite growth, and the domain boundary is still identifiable. Significant crystallite growth was observed at 1100 °C, together with densification of some of the domains via inter-particle sintering to form dense aggregates. The final powder was found to have a specific surface area of ~8.3 m^2^/g, corresponding to an average particle size of ~140 nm. The value is significantly larger than that observed from the FE-SEM micrograph (up to ~90 nm) for the primary particles, due to the presence of hard aggregates. The 800 and 900 °C powders from SO_4_^2−^-L(Y_0.98_Eu_0.02_)H show morphologies similar to their counterparts described above, except that the domains in the 900 °C product are much less disintegrated since the Ln_2_O_2_SO_4_ phase (Fig. 5), finely mixed with the Ln_2_O_3_ portion, significantly restricts crystallite growth. Calcination to 1100 °C causes complete collapse of the domains to yield a substantially better dispersed oxide powder, and the evolution of SO_*x*_ gas was believed to promote disintegration of the domains. Accordingly, the final oxide has a much higher specific surface area of ~17.5 m^2^/g (average particle size ~67 nm). The amount of residual sulfur in the oxide phosphors calcined at 1100 °C was assayed via ICP elemental analysis to be up to 0.18 wt.% in our previous work [50].

**Fig. 8:**
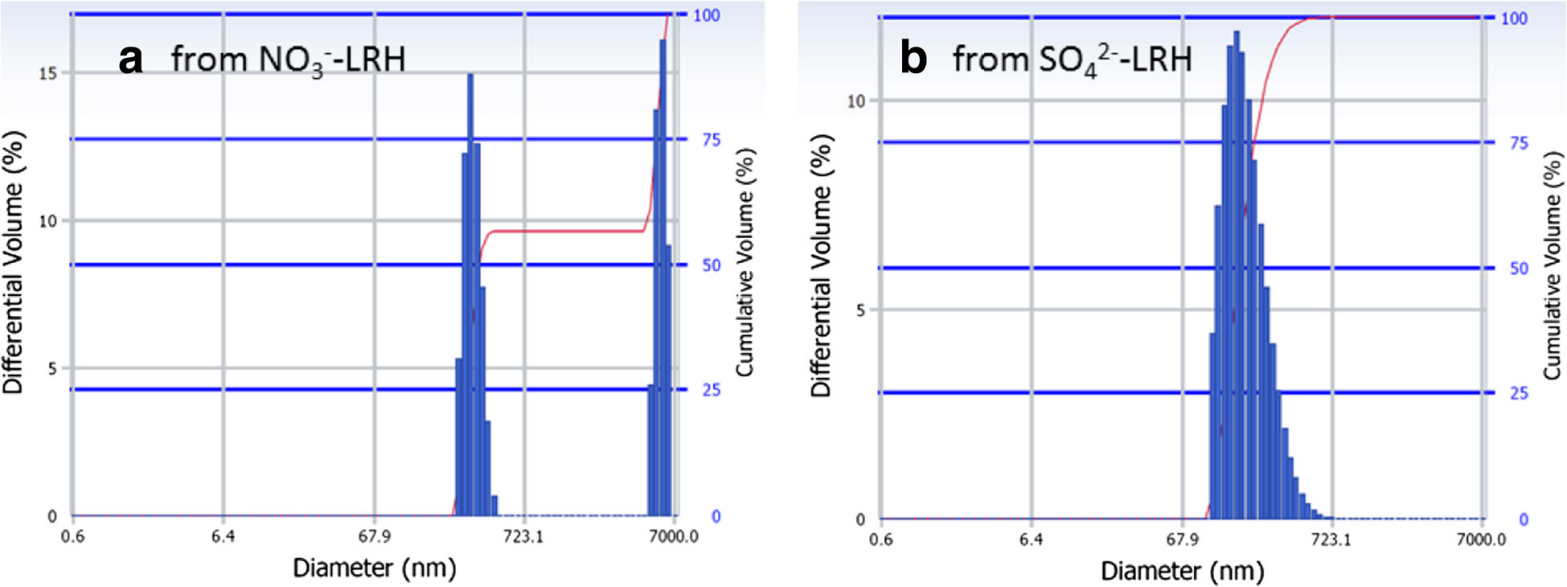
Particle size/size distribution analysis of the (Y_0.98_Eu_0.02_)_2_O_3_ powders. Particle size/size distribution analysis of the (Y_0.98_Eu_0.02_)_2_O_3_ powders calcined at 1100 °C for 4 h from **a** the NO_3_^−^-L(Y_0.98_Eu_0.02_)H and **b** the SO_4_^2−^-L(Y_0.98_Eu_0.02_)H. The size distribution is given in cumulative volume (*right axis*)

Figure 8 shows the particle size/size distribution of the two kinds of (Y_0.98_Eu_0.02_)_2_O_3_ powders calcined at 1100 °C. It is clearly seen that the powder from NO_3_^−^-L(Y_0.98_Eu_0.02_)H exhibits a bimodal size distribution owing to the presence of hard aggregates (Fig. 7). The finer portion (~56.5 vol.%) has an average particle (cluster) size of ~320 ± 43 nm while the coarser part has a value of ~6.21 ± 0.53 μm. A unimodal size distribution was observed for the (Y_0.98_Eu_0.02_)_2_O_3_ powder from SO_4_^2−^-L(Y_0.98_Eu_0.02_)H, and the average particle size was analyzed to be ~219 ± 94 nm. The above results are in agreement with morphology observations (Fig. 7), and further confirm that SO_4_^2−^ exchange of the interlayer NO_3_^−^ is beneficial to the derivation of finer and better dispersed oxide powders.

### Photoluminescent Properties of the (Y_0.98_RE_0.02_)_2_O_3_ Nanophosphors

**Fig. 9:**
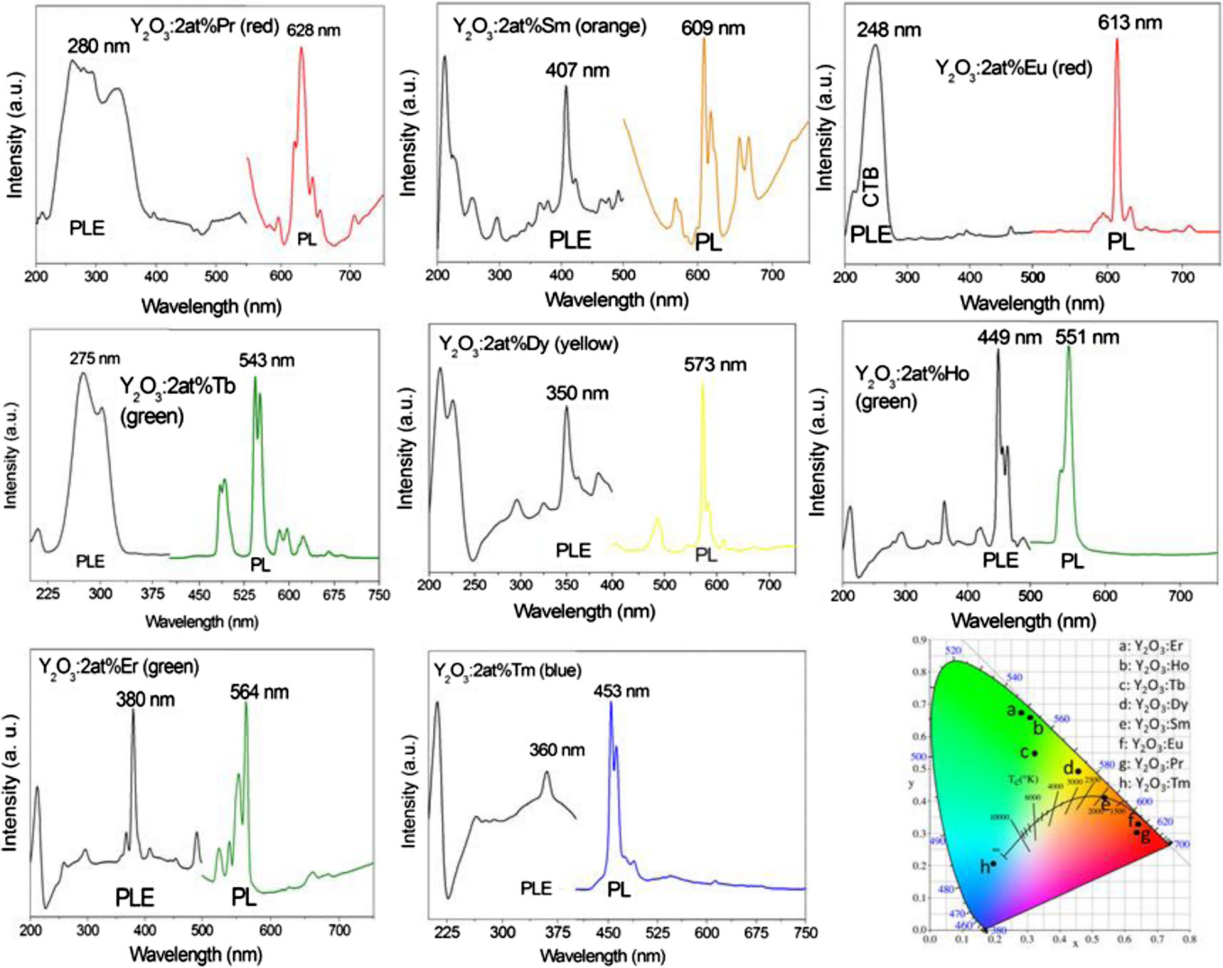
PLE and PL spectra for the (Y_0.98_RE_0.02_)_2_O_3_ nanophosphors. PLE (*left*) and PL (*right*) spectra for the (Y_0.98_RE_0.02_)_2_O_3_ nanophosphors calcined from SO_4_^2−^-LLnH at 1100 °C for 4 h, together with the CIE chromaticity diagram for the emissions

Figure 9 shows photoluminescence excitation/emission spectra for the (Y_0.98_RE_0.02_)_2_O_3_ nanophosphors calcined at 1100 °C, with the excitation and emission wavelengths used for the measurements indicated in each part of the figure. The origins of these main bands [19, 51–53] are summarized in Table 1, together with the chromaticity coordinates of emission and the fluorescence lifetime. The origins of the other PLE/PL bands in each part of Fig. 9 are well documented and can be found in the literature [19, 51–53]. It is seen from the Commission Internationale de l’Eclairage (CIE) chromaticity diagram that the phosphors synthesized in this work span a wide range of emission colors, from blue (Tm^3+^) to deep red (Pr^3+^) via green (Tb^3+^, Ho^3+^, and Er^3+^), yellow (Dy^3+^), orange (Sm^3+^), and orange red (Eu^3+^).

**Table 1:** Optical properties of the (Y_0.98_RE_0.02_)_2_O_3_ nanophosphors

RE	Main PLE band (nm)	Main PL band (nm)	CIE coordinates (*x*,*y*)	Emission color	Lifetime
Pr	280, 4f^2^ → 4f^1^5d^1^	645,^1^D_2_ → ^3^H_4_	(0.639,0.303)	Deep red	160 ± 13 ns
Sm	407, ^6^H_5/2_ → ^4^K_11/2_	609, ^4^G_5/2_ → ^6^H_7/2_	(0.538,0.414)	Orange	1.52 ± 0.01 ms
Eu	250, CTB (O^2-^ → Eu^3+^)	613, ^5^D_0_ → ^7^F_2_	(0.640,0.329)	Orange red	2.71 ± 0.02 ms
Tb	275, 4f^8^ → 4f^7^5d^1^	545, ^5^D_4_ → ^7^F_5_	(0.321,0.551)	Green	3.08 ± 0.02 ms
Dy	350, ^6^H_15/2_ → ^6^P_7/2_	573, ^4^F_9/2_ → ^6^H_13/2_	(0.457,0.492)	Yellow	229 ± 12 ns
Ho	449, ^5^I_8_ → ^5^F_1_	551, ^5^S_2_ → ^5^I_8_	(0.308,0.657)	Green	126 ± 9 ns
Er	380, I_15/2_ → ^4^G_11/2_	564, ^4^S_3/2_ → ^4^I_15/2_	(0.281,0.675)	Green	246 ± 15 ns
Tm	360, ^3^H_6_ → ^1^D_2_	453, ^1^D_2_ → ^3^F_4_	(0.194,0.206)	Blue	170 ± 13 ns

A summary of optical properties of the (Y_0.98_RE_0.02_)_2_O_3_ nanophosphors

## Conclusions

It is shown in this work that coprecipitation at the freezing temperature of ~4 °C can directly produce, without exfoliation, solid-solution nanosheets of the nitrate-type layered hydroxides of Ln_2_(OH)_5_NO_3_·*n*H_2_O (NO_3_-LLnH, Ln = Y_0.98_RE_0.02_, and RE = Pr, Sm, Eu, Tb, Dy, Ho, Er, and Tm). Replacement of the interlayer NO_3_^−^ with SO_4_^2−^ via in situ anion exchange was achieved to produce the sulfate derivative of SO_4_^2−^-LLnH. Detailed characterizations of both the types of layered materials and their calcination products via the combined techniques of XRD, FTIR, DTA/TG, FE-SEM/TEM, BET, particle sizing, and photoluminescence spectroscopy have led to the following main conclusions: (1) anion exchange did not bring about any appreciable change to the layered structure and the two-dimensional crystallite morphology, but induces a basal-spacing contraction from ~0.886 to 0.841 nm, (2) the interlayer SO_4_^2−^ significantly raises the decomposition temperature of the nanosheets from ~600 to 1000 °C to yield oxide via a monoclinic-structured Ln_2_O_2_SO_4_ intermediate phase, and (3) the (Y_0.98_RE_0.02_)_2_O_3_ powders from SO_4_^2−^-LLnH are much better dispersed and finer than those from NO_3_-LLnH, and exhibit emission colors, depending on RE^3+^, covering a wide range in the CIE chromaticity diagram, from blue to deep red via green, yellow, orange, and orange red.

## References

[1] Igarashi T, Ihara M, Kusunoki T, Ohno K (2000). Relationship between optical properties and crystallinity of nanometer Y_2_O_3_:Eu^3+^ phosphor. Appl Phys Lett.

[2] Zhu Q, Li JG, Li XD, Sun XD (2009). Morphology-dependent crystallization and luminescence behavior of (Y, Eu)_2_O_3_ red phosphors. Acta Mater.

[3] Blasse G, Grabmair BC (1994). Luminescent materials.

[4] Hong GY, Jeon BS, Yoo YK, Yoo JS (2001). Photoluminescence characteristics of spherical Y_2_O_3_:Eu^3+^ phosphors by aerosol pyrolysis. J Electrochem Soc.

[5] Ekambaram S, Patil KC, Mazza M (2005). Synthesis of lamp phosphors: facile combustion approach. J Alloys Compd.

[6] Kim EJ, Kang YC, Park HD, Ryu SK (2003). UV and VUV characteristics of (YGd)_2_O_3_:Eu phosphor particles prepared by spray pyrolysis from polymeric precursors. Mater Res Bull.

[7] Lenggoro IW, Itoh Y, Okuyama K, Kim TO (2004). Nanoparticles of a doped oxide phosphor prepared by direct-spray pyrolysis. J Mater Res.

[8] Wakefield G, Holland E, Dobson PJ, Hutchison JL (2001). Luminescence properties of nanocrystalline Y_2_O_3_:Eu^3+^. Adv Mater.

[9] Camenzind A, Strobel R, Pratsinis SE (2005). Cubic or monoclinic Y_2_O_3_:Eu^3+^ nanoparticles by one step flame spray pyrolysis. Chem Phys Lett.

[10] Li J-G, Ishigaki T (2012). One-step Ar/O_2_ thermal plasma processing of Y_2_O_3_:Eu^3+^ red phosphors: phase structure, photoluminescent properties, and the effects of Sc^3+^ codoping. J Solid State Chem.

[11] Li J-G, Li XD, Sun XD, Ishigaki T (2008). Monodispersed colloidal spheres for uniform Y_2_O_3_:Eu^3+^ red-phosphor particles and greatly enhanced luminescence by simultaneous Gd^3+^ doping. J Phys Chem C.

[12] Zhu Q, Li J-G, Li XD, Sun XD, Sakka Y (2011). Monodisperse colloidal spheres for (Y, Eu)_2_O_3_ red-emitting phosphors: establishment of processing window and size-dependent luminescence behavior. Sci Technol Adv Mater.

[13] Wu CF, Qin WP, Qin GS (2003). Photoluminescence from surfactant-assembled Y_2_O_3_:Eu^3+^ nanotubes. Appl Phys Lett.

[14] Zhang JL, Hong GY (2004). Synthesis and photoluminescence of the Y_2_O_3_:Eu^3+^ phosphor nanowires in AAO template. J Solid State Chem.

[15] Wan JX, Wang ZH, Chen XY, Mu LY, Qian YT (2005). Shape-tailored photoluminescent intensity of red phosphor Y_2_O_3_:Eu^3+^. J Cryst Growth.

[16] He Y, Tian Y, Zhu ZF (2003). Large-scale synthesis of luminescent Y_2_O_3_:Eu nanobelts. Chem Lett.

[17] Wu X, Tao Y, Gao F, Dong L, Hu Z (2005). Preparation and photoluminescence of yttrium hydroxide and yttrium oxide doped with europium nanowires. J Cryst Growth.

[18] Lu ZG, Qian DY, Tang G (2005). Facile synthesis and characterization of sheet-like Y_2_O_3_: Eu^3+^ microcrystals. J Cryst Growth.

[19] Lin J, Yu M, Lin CK, Liu XM (2007). Multiform oxide optical materials via the versatile Pechini-type sol-gel process: synthesis and characteristics. J Phys Chem C.

[20] Gándara F, Perles J, Snejko N, Iglesias M, Gómez-Lor B, Gutiérrez-Puebla E (2006). Layered rare-earth hydroxides: a class of pillary crystalline compounds for intercalation chemistry. Angew Chem Int Ed.

[21] Geng FX, Matsushita Y, Ma R, Xin H, Tanaka M, Izumi F, Iyi N, Sasaki T (2008). General synthesis and structural evolution of a layered family of Ln_8_(OH)_20_C_l4_·*n*H_2_O (Ln = Nd, Sm, Eu, Gd, Tb, Dy, Ho, Er, Tm, and Y). J Am Chem Soc.

[22] Geng FX, Xin H, Matsushita Y (2008). New layered rare-earth hydroxides with anion-exchange properties. Chem Eur J.

[23] Geng FX, Matsushita Y, Ma R (2008). General synthesis and structural evolution of a layered family of Ln_8_(OH)_20_C_l4_·*n*H_2_O (Ln = Nd, Sm, Eu, Gd, Tb, Dy, Ho, Er, Tm, and Y). Inorg Chem.

[24] Geng FX, Ma R, Sasaki T (2010). Anion-exchangeable layered materials based on rare-earth phosphors: unique comination of rare-earth host and exchangeable anions. Acc Chem Res.

[25] McIntyre LJ, Jackson LK, Fogg AM (2008). Synthesis and anion exchange chemistry of new intercalation hosts containing lanthanide cations, Ln_2_(OH)_5_NO_3_·*n*H_2_O (Ln = Y, Gd-Lu). J Phys Chem Solids.

[26] Poudret L, Prior TJ, McIntyre LJ (2008). Synthesis and crystal structures of new lanthanide hydroxyhalide anion exchange materials, Ln_2_(OH)_5_X·1.5H_2_O (X = Cl, Br; Ln = Y, Dy, Er, Yb). Chem Mater.

[27] McIntyre LJ, Jackson LK, Fogg AM (2008). Ln_2_(OH)_5_NO_3_·*x*H_2_O (Ln = Y, Gd-Lu): a novel family of anion exchange intercalation hosts. Chem Mater.

[28] Hindocha SA, McIntyre LJ, Fogg AM (2009). Precipitation synthesis of lanthanide hydroxyl nitrate anion exchange materials, Ln_2_(OH)_5_NO_3_·H_2_O (Ln = Y, Eu-Er). J Solid State Chem.

[29] McIntyre LJ, Prior TJ, Fogg AM (2010). Observation and isolation of layered and framework ytterbium hydroxide phases using in situ energy-dispersive X-ray diffraction. Chem Mater.

[30] Lee K-H, Byeon S-H (2009). Extended menberd of the layered rare-earth hydroxides family, RE_2_(OH)_5_NO_3_·*n*H_2_O (RE = Sm, Eu, and Gd): synthesis and anion-exchange behavior. Eur J Inorg Chem.

[31] Lee K-H, Byeon S-H (2009). Synthesis and aqueous colloidal solutions of RE_2_(OH)_5_NO_3_·*n*H_2_O (RE = Nd and La). Eur J Inorg Chem.

[32] Zhu Q, Li J-G, Zhi CY, Ma RZ, Sasaki T, Xu JX, Liu CH, Li XD, Sun XD, Sakka Y (2011). Nanometer-thin layered hydroxide platelets of (Y_0.95_Eu_0.05_)_2_(OH)_5_NO_3_·*x*H_2_O: exfoliation-free synthesis, self-assembly, and the derivation of dense oriented oxide films of high transparency and greatly enhanced luminescence. J Mater Chem.

[33] Zhu Q, Li J-G, Zhi CY, Li XD, Sun XD, Sakka Y, Golberg D, Bando Y (2010). Layered rare-earth hydroxides (LRHs) of (Y_1-*x*_Eu_*x*_)_2_(OH)_5_NO_3_·*n*H_2_O(*x* = 0-1): structural variations by Eu^3+^ doping, phase conversion to oxides, and the correlation of photoluminescence behaviors. Chem Mater.

[34] Zhu Q, Li J-G, Ma RZ, Sasaki T, Yang XJ, Li XD, Sun XD, Sakka Y (2012). Well-defined crystallites autoclaved from the nitrate/NH_4_OH reaction system as the precursor for (Y, Eu)_2_O_3_ red phosphor: crystallization mechanism, phase and morphology control, and luminescent property. J Solid State Chem.

[35] Hu LF, Ma RZ, Ozawa TC, Sasaki T (2010). Exfoliation of layered europium hydroxide into unilamellar nanosheets. Chem Asian J.

[36] Lee KH, Lee BI, You JH, Byeon SH (2010). Transparent Gd_2_O_3_:Eu phosphor layer derived exfoliated layered gadolinium hydroxide nanosheets. Chem Commun.

[37] Yoon YS, Byeon SH, Lee IS (2010). Unexplored thermal transformation behavior of two- dimensionally bound gadolinium hydroxide layers: fabrication of oriented crystalline films of gadolinium oxychloride nanosheets suitable for the multicolor luminescence with color tenability. Adv Mater.

[38] Lee BI, Lee ES, Byeon SH (2012). Assembly of layered rare-earth hydroxide nanosheets and SiO_2_ nanoparticles to fabricate multifunctional transparent films capable of combinatorial color generation. Adv Funct Mater.

[39] Zhu Q, Li JG, Li XD, Sun XD, Qi Y, Zhu MY (2014). Tens of micron sized unilamellar nanosheets of Y/Eu layered rare-earth hydroxide (LRH): efficient exfoliation via fast anion exchange and their self-assembly into oriented oxide film with enhanced photoluminescence. Sci Technol Adv Mater.

[40] Wu XL, Li J-G, Zhu Q, Li JK, Ma R, Sasaki T, Li XD, Sun XD, Sakka Y (2012). The effects of Gd^3+^ substitution on the crystal structure, site symmetry, and photoluminescence of Y/Eu layered rare-earth hydroxide (LRH) nanoplates. Dalton Trans.

[41] Wu XL, Li J-G, Li JK, Zhu Q, Li XD, Sun XD (2013). Layered rare-earth hydroxide (LRH) and oxide nanoplates of the Y/Tb/Eu system: phase controlled processing, structure characterization, and color-tunable photoluminescence via selective excitation and efficient energy transfer. Sci Technol Adv Mater.

[42] Wu XL, Li J-G, Ping D-H, Li JK, Zhu Q, Li XD, Sun XD, Sakka Y (2013). Structure characterization and photoluminescence properties of (Y_0.95-*x*_Gd_*x*_Eu_0.05_)_2_O_3_ red phosphors converted from layered rare-earth hydroxide (LRH) nanoflake precursors. J Alloys Compd.

[43] Wu XL, Li J-G, Zhu Q, Liu WG, Li J, Li XD, Sun XD, Sakka Y (2015). One-step freezing temperature crystallization of layered rare-earth hydroxide (Ln_2_(OH)_5_NO_3_·*n*H_2_O) nanosheets for a wide spectrum of Ln (Ln = Pr-Er, and Y), anion exchange with fluorine and sulfate, and microscopic coordination probed via photoluminescence. J Mater Chem C.

[44] Nakamoto K (1963). Infrared spectra of inorganic and coordination compounds.

[45] Gadsden JA (1975). Infrared spectra of minerals and related inorganic compounds.

[46] Shannon RD (1976). Revised effective ionic-radii and systematic studies of interatomic distances in halides and chalcogenides. Acta Crystallogr Sect A Cryst Phys Diffr Theor Gen Crystallogr.

[47] Liang JB, Ma R, Geng FX, Ebina Y, Sasaki T (2010). Ln_2_(OH)_4_SO_4_·*n*H_2_O (Ln = Pr to Tb; *n*~2): a new family of layered rare-earth hydroxides rigidly pillared by sulfate ions. Chem Mater.

[48] Wang XJ, Li J-G, Zhu Q, Li XD, Sun XD, Sakka Y (2014). Facile and green synthesis of (La_0.95_Eu_0.05_)_2_O_2_S red phosphors with sulfate-ion pillared layered hydroxides as a new type of precursor: controlled hydrothermal processing, phase evolution and photoluminescence. Sci Technol Adv Mater.

[49] Wang XJ, Li J-G, Zhu Q, Li XD, Sun XD, Sakka Y (2014). Synthesis, characterization, and photoluminescent properties of (La_0.95_Eu_0.05_)_2_O_2_SO_4_ red phosphors with layered hydroxyl sulfate as precursor. J Alloys Compd.

[50] Lu B, Li J-G, Suzuki TS, Estili M, Liu WG, Sun XD, Sakka Y (2015). Controlled synthesis of layered rare-earth hydroxide nanosheets leading to highly transparent (Y_0.95_Eu_0.05_)_2_O_3_ ceramics. J Am Ceram Soc.

[51] Li J-G, Sakka Y (2015). Recent progress in advanced optical materials based on gadolinium aluminate garnet (Gd_3_Al_5_O_12_). Sci Technol Adv Mater.

[52] Yang J, Li C, Quan Z, Zhang C, Yang P, Li Y, Yu C, Lin J (2008). Self-assembled 3D flowerlike Lu_2_O_3_ and Lu_2_O_3_:Ln^3+^ (Ln = Eu, Tb, Dy, Pr, Sm, Er, Ho, Tm) microarchitectures: ethylene glycol-mediated hydrothermal synthesis and luminescent properties. J Phys Chem C.

[53] Aumuller GC, Kostler W, Grabmaier BC, Grey R (1994). Luminescence properties of Pr^3+^ in cubic rare-earth-oxides. J Phys Chem Solids.

